# Validating a work group climate assessment tool for improving the performance of public health organizations

**DOI:** 10.1186/1478-4491-3-10

**Published:** 2005-10-13

**Authors:** Cary Perry, Nancy LeMay, Greg Rodway, Allison Tracy, Joan Galer

**Affiliations:** 1 Management & Leadership Program, Management Sciences for Health, Cambridge, Massachusetts, USA; 2 Wellesley Centers for Women, Wellesley College, Wellesley, Massachusetts, USA

## Abstract

**Background:**

This article describes the validation of an instrument to measure work group climate in public health organizations in developing countries. The instrument, the Work Group Climate Assessment Tool (WCA), was applied in Brazil, Mozambique, and Guinea to assess the intermediate outcomes of a program to develop leadership for performance improvement. Data were collected from 305 individuals in 42 work groups, who completed a self-administered questionnaire.

**Methods:**

The WCA was initially validated using Cronbach's alpha reliability coefficient and exploratory factor analysis. This article presents the results of a second validation study to refine the initial analyses to account for nested data, to provide item-level psychometrics, and to establish construct validity. Analyses included eigenvalue decomposition analysis, confirmatory factor analysis, and validity and reliability analyses.

**Results:**

This study confirmed the validity and reliability of the WCA across work groups with different demographic characteristics (gender, education, management level, and geographical location). The study showed that there is agreement between the theoretical construct of work climate and the items in the WCA tool across different populations. The WCA captures a single perception of climate rather than individual sub-scales of clarity, support, and challenge.

**Conclusion:**

The WCA is useful for comparing the climates of different work groups, tracking the changes in climate in a single work group over time, or examining differences among individuals' perceptions of their work group climate. Application of the WCA before and after a leadership development process can help work groups hold a discussion about current climate and select a target for improvement. The WCA provides work groups with a tool to take ownership of their own group climate through a process that is simple and objective and that protects individual confidentiality.

## Background

This article describes the validation of an instrument to measure work group climate in public health organizations in developing countries. In light of decentralizing health care systems and the urgent need to scale up services to combat HIV/AIDS, tuberculosis, and malaria, it is critical that providers of technical assistance have valid tools to help build institutional capacity in both public-sector and nongovernmental organizations. Health care managers in developing countries need a simple, inexpensive tool that is objective and applicable to small work groups and that can become part of the team's own self-evaluation process. The goal of improving work group climate is to strengthen organizational performance and improve health service delivery.

The development of the Work Group Climate Assessment Tool was carried out between 2002 and 2004 by the Management & Leadership (M&L) Program, a five-year cooperative agreement between the United States Agency for International Development (USAID) and Management Sciences for Health (MSH). MSH is a nonprofit organization with headquarters in Cambridge, Massachusetts. M&L works with ministries of health, national and international programs, and nongovernmental organizations in 27 developing countries to strengthen the leadership skills of health personnel and the management systems that are essential to deliver high-quality health services.

A positive work group climate is a primary outcome of a leadership development process aimed at improving the performance of managers and their work groups. This hypothesis is based on evidence that leadership and management practices that provide employees with clarity, support, and challenge contribute to a positive work climate. A positive work climate leads to and sustains employee motivation and high performance by liberating "discretionary effort," or the level of extra effort that employees exert above and beyond job expectations [1].

### Organizational climate and culture

The terms "organizational climate" and "organizational culture" are sometimes treated as different concepts because they arise out of distinct theoretical traditions, but they have many overlapping elements. Stringer explains climate as a subset of organizational culture [2]. Culture applies to the deeply rooted value systems inherent in all organizations and is difficult to change [3]. Organizational strategy, the external environment, organizational arrangements and historical forces all affect the context and milieu within which a work group operates. These "cultural" influences develop outside the work group and are beyond the direct control of the work group manager. Burke describes the relationship between climate and culture in a series of papers that discuss the modification of organizational culture at British Airways [4,5]. Burke posits that changes to climate are more achievable than changes in culture, because climate is associated with the "transactional level of human behaviour – the everyday interactions and exchanges" [4].

Thus, while every organization has an organizational *culture*, each work group (or team) within the organization has its particular *climate*. Tagiuri defines organizational climate as a "quality of the internal environment of an organization that (a) is experienced by its members, (b) influences their behaviour and (c) can be described in terms of the values of a particular set of characteristics (or attributes) of the organization" [6]. Burke [4] stresses that leadership, mission, strategy and organizational culture have an organization-wide focus, whereas climate is experienced and created in the work group or team.

A work group's climate may be similar to or different from the overall organizational climate. High-performing work groups sometimes operate in organizations troubled by declining funding or inadequate leadership at the senior level. The leadership and management practices of a manager can create a positive work climate and strong results within a work group, even if an organization's climate is less than optimal. Regardless of a manager's level, his or her efforts to improve the work group's climate can contribute to strong employee performance and results.

### Work group climate and performance

Positive work climate has been identified in a variety of environments as a driver of performance. According to the business literature, there is a positive correlation between climate and performance and also between climate and financial results. "Organizational climate is not the only driver of performance. Economic conditions and competitive dynamics matter enormously. But our analysis suggests that climate accounts for nearly a third of the results" [1].

While many of the factors such as an organization's history, culture, organizational strategies and structures are outside the control of the work group manager, the manager is uniquely placed to influence work climate within the work group. "What the boss of a work group does is the most important determinant of climate. The boss's behavior drives climate, which arouses motivation. And aroused motivation is a major driver of bottom-line performance" [2].

The work group manager, therefore, has a substantial impact on the development of work climate and the productivity of the work group. This relationship was clearly identified by a research project conducted at Harvard Business School in 1968. The project studied the relationship between motivation and organizational climate and reviewed the impact of different leadership styles on three evenly matched teams working on the same production project. The researchers demonstrated that leadership styles affected both the development of work group climate and the productivity of the three teams [7].

The impact of work climate is not restricted to the commercial sector. Research in the health and education fields supports the conclusions from the business literature. For instance, in a study of Canadian staff nurses, Laschinger, Finegan and Shamian describe the relationship between empowerment, job satisfaction, and commitment [8]. A positive work climate creates an environment conducive to the development of trust and empowerment, which in turn leads to high-quality patient care [8].

Positive climate has also been demonstrated to drive success in schools operated by the Department of Education and Employment in the United Kingdom. "Our research demonstrates a significant link between classroom climate and student academic progress. . . to the degree that teachers can develop skills and characteristics that impact climate, so they can hope to more effectively motivate and engage their students" [9].

Over the past decade a number of instruments have been developed to help organizations measure organizational climate, predominantly in US-based organizations in the private sector. However, these tools are often proprietary (and therefore expensive) or complicated and lengthy to administer. Surveys of organizational climate such as the PAHO Organizational Climate Instrument [10], and the Gallup Q12 [11] do not conform to the needs of small work groups. The 80-item PAHO Organizational Climate Instrument is extremely lengthy, and like the Q12, measures individual employees' satisfaction, rather than perception of the overall climate in a work group.

## Methods

### Description of the Work Group Climate Assessment Tool

The Work Group Climate Assessment Tool (WCA) is a self-administered assessment form originally consisting of 14 items: 12 that correspond to three sub-dimensions of climate – clarity, support and challenge – and 2 items that capture perceptions of productivity and quality. These sub-dimensions and the individual items are based on the work of George Litwin and Robert Stringer, who pioneered the study of climate in corporate environments [2,7].

The WCA is designed to measure climate among intact teams or work groups in the health sector of developing countries. (An intact team is defined as a group of individuals who work together regularly at the same work site, whether in a central or regional office or a health facility.) The WCA is the first assessment tool that has been developed for this purpose. It is intended to measure climate in work groups at any level of an organization. To date, the WCA has been used to measure work group climate before and after the M&L Leading for Performance Improvement Program that was conducted in five sites – Egypt, Mozambique, Brazil, Guinea, and Kenya – and via a virtual distance-learning program for leadership development.

The WCA is divided into two sections. The first section includes 12 items, which were mapped to the three hypothesized sub-dimensions mentioned above of clarity, support and challenge. The items for the original WCA were the following:

1. We are recognized for individual contributions.

2. We have a common purpose.

3. We have the resources we need to do our jobs well.

4. We develop our skills and knowledge.

5. We have a plan which guides our activities.

6. We strive to improve our performance.

7. We understand each other's capabilities.

8. We are clear about what is expected in our work.

9. We seek to understand the needs of our clients.

10. We participate in the decisions of our work group.

11. We take pride in our work.

12. We readily adapt to new circumstances.

The second section, items 13 and 14, relates to perceptions of productivity and quality, which are defined for the respondent on the assessment form:

13. Our work group is known for quality work.

14. Our work group is productive.

To apply the survey, all members of the work group (both managerial and staff) complete the assessment form. Each team member rates each item. The scores are then tabulated across all respondents, and results for each item and an overall climate score for items 1–12 are calculated for the team as a whole. Results for items 13 and 14 are calculated separately.

At the conclusion of the leadership program, the WCA is applied again among all team members. The post-intervention scores are again calculated for the team as a whole and then compared to the baseline team scores and targets to determine the amount of change produced by the intervention vis-à-vis the anticipated results (climate targets).

### Initial validation of the WCA

M&L tested the WCA for face validity throughout 2002–2003 with counterparts in Brazil and Nicaragua as well as with several teams working on the M&L Program in Cambridge. In addition, the WCA was used to collect baseline and follow-up data among participants in the Leading for Performance Improvement Programs in Egypt and Guinea and participants in the Virtual Leadership Development Program in Latin America. The WCA was translated into Portuguese, Spanish and French and pre-tested in the different countries to make sure that it was appropriate for use across cultures. Based on feedback from the field tests, the WCA form and instructions were refined, and ultimately published in 2003 [12]. The expectation was that publishing the tool would allow M&L leadership programs to test and refine the tool in preparation for a full validation study and peer-review.

Based on the work of Litwin and Stringer [7], the original tool incorporated a measure of the importance of each item to the respondent in order to assist teams to prioritize the sub-dimensions that needed more attention. Managers participating in the pre-testing of the instrument, however, tended to rate the importance of all items quite high, and therefore the importance measure was not useful for determining the teams' priorities. Part of the purpose of the current study was to determine if the importance column could be eliminated without compromising the statistical validity of the tool.

Using the data collected during the field tests, M&L examined certain aspects of the tool's validity and reliability. For example, data from Brazil suggest that the tool has discriminant validity. The WCA was applied with three groups of managers in Brazil: one group was in the state of Ceará and had undergone extensive leadership training over a period of five years, while the other two groups were in states that had only begun to participate in leadership training. The spread in mean scores from a high mean in Ceará to much lower means in the other two states suggests the tool can discriminate between high- and low-performing work groups.

In terms of reliability, a Cronbach's alpha of 0.87 was calculated on 122 cases of WCA data collected in Latin America and Egypt. The coefficient alpha suggests that the items in the WCA have a high level of internal consistency. Results of an initial factor analysis conducted on the same data indicate that the assessment items load on a set of three to four factors. However, additional analysis on a larger data set was necessary to determine whether the identified factors relate to the hypothesized sub-dimensions of clarity, challenge and support.

While the WCA was initially validated using Cronbach's alpha reliability coefficient and exploratory factor analysis, a subsequent validation study was necessary to refine these analyses to account for nested data, provide item-level psychometrics and establish construct validity. This paper presents the results of this second validation study. Study participants were a purposive sample of present and past recipients of M&L technical assistance to strengthen management and leadership in the public health sector in developing countries. Participants came from ministries of health in Mozambique and Guinea, the Secretariat of Health for the State of Ceará, Brazil, and Brazilian public health laboratories. The participants represented a wide variety of positions, including central-level ministry staff, district-level managers, hospital administrators, laboratory technicians and clinic personnel.

The participants completed self-administered questionnaires anonymously in a group setting in each participating organization in May 2004. The survey contained two sections: The first consisted of the original 12 climate items from the WCA, the two productivity and quality items, and nine additional items generated to increase the item pool for measurement refinement. The second section consisted of 24 items from one section of the Stringer Organizational Climate Survey [7]. Participants rated each item on a Likert scale, where 1 = not at all, 2 = to a small degree, 3 = to a moderate degree, 4 = to a great degree and 5 = to a very great degree.

The Stringer survey served as the gold standard for this study. This instrument, which has been used repeatedly since 1968, was validated through studies that showed its association with objective measures of organizational climate in corporate settings in the United States [2].

The analysis data set consisted of data from 305 individuals in 42 work groups: Brazil (21 work groups, 182 employees), Mozambique (18 work groups, 97 employees), and Guinea (3 work groups, 26 employees). With 42 work group sites, we had a statistical power of 0.87 to detect validation correlations as low as 0.20 at the standard significance value of 0.05. Values outside the admissible range for a given variable were reassigned as missing. Four cases were omitted from the analysis sample due to miskeyed data.

Analyses included eigenvalue decomposition analysis, confirmatory factor analysis (which included tests for measurement noninvariance by gender, management status and educational level), and construct validity and reliability analyses of the WCA. Once a final set of items had been selected, reliability coefficients for both the work group and individual employee levels of analysis were computed.

## Results and discussion

### Description of participants

Among participants from Brazil and Mozambique, women represented 62% of the respondents, consistent with trends of female participation in the health field. In these two samples, 59 respondents were managers (22%). Individuals had been employed in the health field for up to 40 years (mean [M] = 17.7, standard deviation [SD] = 9.7) and in the rated organization for up to 37 years (M = 11.5, SD = 9.2). Demographic information was not available for the three Guinean work groups.

### Item weighting

Respondents to the WCA survey rate each item twice: first according to how the item was currently performed in the work group ("actual performance") and second according to the item's perceived importance to the work group. Models using the "actual performance" ratings were compared to those weighted by "importance" ratings. (A weighted score is the value assigned to the "actual performance" of a given item multiplied by the value of the "importance" assigned to that item.) It was found that in the importance-weighted models the factor structure was stronger, due to improved distributional characteristics of the individual items. As a result, all subsequent models in the study used importance-weighted scores for the WCA items. However, use of the WCA in the field suggests that the weighting of actual scores by importance scores is not easily understood by health managers and is not useful for prioritizing actions to improve climate. To determine if the weighting could be eliminated, we correlated mean individual scores from the "actual performance" ratings and those same scores weighted by the "importance" rating. This correlation was extremely high (R = 0.83, *p *< 0.01), indicating that the importance column could be eliminated without compromising the validity of the tool.

### Eigenvalue decomposition analysis

**Figure 1:**
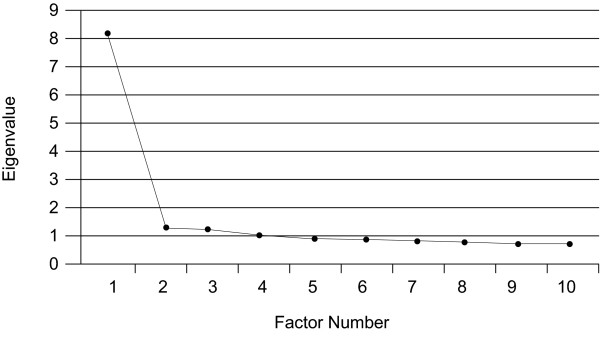
Scree plot of eigenvalues (importance-weighted WCA items) for the full sample.

The variance/covariance matrix of the 21 items from the WCA were submitted to an eigenvalue decomposition analysis, and the resulting eigenvalues were plotted (Fig. 1). (Items 13–14, measuring productivity and quality, were not included in the model and were analysed separately, since they are considered outputs of a well-functioning work group rather than components of climate.) The resulting scree plot clearly shows a unidimensional structure. Scree plots based on eigenvalues obtained from each of the three separate countries (not shown) also indicated a clearly unidimensional construct. Initially, models were conducted by disaggregating the items into the three hypothesized sub-dimensions: clarity, challenge, and support. While the factor loadings were strong for all three sub-dimensions, the factor intercorrelations were very high (0.81 to 0.95), and modification indices suggested that there was considerable cross-loading of items across factors. Because of a lack of factor discrimination and because the scree plot (Fig. 1) shows evidence of a single dimension, all subsequent models contain a single WCA factor.

### Confirmatory factor analyses

An initial confirmatory factor model with the full set of 21 importance-weighted items was fitted to adjust for the clustering in the data. In this model and all subsequent models, missing data were handled by means of a maximum likelihood estimation technique, which minimizes bias due to listwise deletion [13]. Factor loadings from this model ranged from 0.37 to 0.68. One item had a factor loading below the standard 0.40 cutoff level [14] so it was omitted from further analysis models.

Noninvariance, or differential item functioning, across various individual characteristics (gender, management status and educational level) was tested by means of a series of multiple group confirmatory factor models in which factor loadings were constrained to be equal across groups. Using a cutoff of 1.00 and above for model-generated modification indices, we identified six items showing evidence of noninvariance across gender, three items showing noninvariance across management status, and three items showing noninvariance across educational levels. (Because there were only 16 respondents with only a primary school educational level, too few to allow estimation, we compared the groups that had finished secondary school [n = 81] and those who had completed university education [n = 160]).

A two-level confirmatory factor model was fitted to 12 importance-weighted WCA items that had at least a 0.40 factor loading in the previous models and that showed no evidence of noninvariance across gender, management status, or educational level. At the work group level of the model, the factor structure of employees' ratings, aggregated to the work group level, was examined for consistency across the 42 work groups. At the individual level of the model, the factor structure of the ratings was examined for consistency across employees *within each work group*. The factor loadings were constrained to be equal across these two levels of analysis so that a parallel interpretation of work group climate was maintained when considering each level of analysis.

At the work group level of the model, there was too little variability in three items to adequately model their contribution to the overall work group climate factor. A fourth item returned a lower than acceptable factor loading (< 0.40). The final model was based on the remaining eight items that showed strong and consistent psychometric properties throughout the preceding series of models. These items and their associated factor loadings are given in Table 1. There was significant variability in the work group climate factor at both the work group (σ = 3.54, *p *< 0.001) and individual (σ = 14.12, *p *< 0.001) levels of analysis, indicating that variability in both work group characteristics and individual employee characteristics may influence overall ratings of work group climate. Rather than eliminate the effects of individual variation, its presence was statistically modeled and the variance partialed into variability that was due to differences between work groups and within the work group across individuals.

**Table 1:** Standardized factor loadings for the final eight WCA items, by level of analysis

	**Individual level**	**Work group level**
4. We feel our work is important.^a^	0.62	0.82
7. We strive to achieve successful outcomes.^a^	0.67	0.90
8. We have a plan which guides our activities.^b^	0.47	0.73
9. We pay attention to how well we are working together.^a^	0.51	0.77
11. We understand each other's capabilities.^b^	0.49	0.79
14. We seek to understand the needs of our clients.^b^	0.66	0.93
15. We understand the relevance of the job of each member in our group.^a^	0.54	0.92
17. We take pride in our work.^b^	0.61	0.93

Notes: ^a ^Additional items included in the model

^b ^Original WCA items

### Validation and reliability analyses

Once the factor model had been finalized, correlations between the WCA and a composite of the Stringer items were estimated at both the individual and work group levels. At the work group level, this correlation was extremely high (R = 0.93, *p *< 0.001), indicating that the eight-item WCA scale captured the same construct as the 24-item Stringer scale in differentiating the work group climate quality across work groups. The correlation at the individual level was moderate (R = 0.48, *p *< 0.001), indicating that the WCA scale captures a construct that is similar to but different from that captured by the Stringer scale in differentiating among employees' perceptions of climate *within *the same work group. The ways in which the WCA differs from the Stringer scale in assessing individual perceptions of the same work group climate require further study to be fully understood.

A reliability analysis of the data on individuals was conducted, without adjusting for the clustered design. The internal consistency in the 8 WCA items across individuals was good (α = 0.81). Since this scale is most commonly used to assess the work group itself, the data were restructured and a reliability analysis using the work group as the level of analysis was done. The internal consistency of the eight items across work groups (aggregated across employees within a work group) was also good (α = 0.86). Our final models contained eight items. Considering only the 42 work groups, this number gives us a ratio of more than five observations per factor, satisfying a common rule of thumb in psychometrics. Additionally, since the estimates of these items at the 42 sites draw from data on 305 individuals, these estimates are very stable, reliable and unbiased.

## Conclusion

The results of this study confirmed the construct validity and reliability of a revised version of the WCA tool across work groups with different demographic characteristics (gender, education, management level and geographical location). This study shows that there is agreement between the theoretical construct of work climate and the items in the WCA tool across different populations. Of the 21 items tested, eight were selected that conferred the greatest measurement power for the smallest number of questions. These items showed the least variance across the different groups and the strongest psychometric properties. The internal consistency of the eight-item WCA was high across work groups, indicating that the individual items in the instrument are associated with each other and all appear to be measuring the same underlying construct. Finally, the eight items selected for the final model correlated well with the 24 items from the Stringer gold standard instrument, indicating that the WCA scale captures the same underlying construct as the Stringer scale in differentiating the climates of work groups.

The WCA was designed to measure three sub-dimensions of climate: clarity, support and challenge. However, study results indicated the WCA items do not discriminate between sub-dimensions of work group climate but rather capture a single perception of climate. While the terms clarity, support and challenge are not measured as sub-dimensions of climate by the WCA tool, they work together to define the construct of the overall quality of work group climate.

Based on these analyses, we recommend that the analysis and feedback of WCA scores to work groups be revised. First, we recommend that the importance column in the tool be eliminated to simplify it further. Second, we recommend that feedback to work groups be provided based on average individual and work group scores and on patterns across the work group (all high scores, all low scores, or a variable pattern of some members reporting positive feelings about climate in the work group while others are reporting more negative feelings). The aspects of clarity, support and challenge are useful to help work groups to discuss their climate, but because the study did not confirm that they exist as separate subscales, scores on individual items or sub-dimensions should not be compared over time.

Once the individual and work group composite scores have been obtained, comparisons can be made between work groups in an organization, between pre- and post-test assessments of the same work group, or between a single work group and a pre-determined value of climate quality that serves as a benchmark. In addition, disparities in the experience of climate within a work group can be assessed by comparing individuals' scores within the work group and tracking changes over time in individuals' perceptions of climate.

Work groups in developing countries can benefit from using the WCA tool as a basis for discussing and improving their climate. Applying the WCA is a simple and rapid exercise that provides a work group with a set of objective scores it can use to select a target for improving climate and to identify strategies for meeting that target. The manager and members of a work group should review their baseline scores (both individual and group) and discuss what may cause the patterns in the data by reflecting on the leading and managing practices used by the group. This process allows the work group's members to decide how they will work together to create a more positive climate.

The WCA also serves as a monitoring tool; once a work group has implemented actions to improve climate and has reapplied the tool, members can compare baseline and follow-up scores to determine what progress they have made in changing their climate. A detailed guide to facilitating the use of the WCA in the field as well as a tabulation sheet, guidelines for analysing and using results, and the tool itself can be downloaded from the MSH website [15].

The results of this study suggest that the WCA is a simple, reliable, and valid instrument for measuring climate among work groups in the health sector of developing countries. This tool is an important contribution to programs working to strengthen the performance of managers and their work groups in order to improve the delivery of health services in developing countries.

## Competing interests

The author(s) declare that they have no competing interests.

## Authors' contributions

These authors contributed equally to this work: Cary Perry, Nancy LeMay, Greg Rodway and Allison Tracy. Cary Perry, Nancy LeMay and Greg Rodway participated in the design of the study, analysis of the data and drafting of the manuscript. Allison Tracy performed the statistical analyses and drafted the results section. Joan Galer conceived of the study and helped develop the instrument. All authors read and approved the final manuscript.
